# Response to the Letter to the Editor Entitled “IMPACT CKD – All Roads Must Not Lead to Dialysis”

**DOI:** 10.1016/j.ekir.2025.06.026

**Published:** 2025-06-16

**Authors:** Juan J. Garcia Sanchez, Stacey Priest, Hannah Guiang, Anthony Zara, David C. Wheeler, Ana F. Moura, Charlotte Johnston-Webber, Jieling Chen

**Affiliations:** 1 Global Market Access & Pricing, AstraZeneca, Barcelona, Spain; 2 Value & Evidence, EEVERSANA, Burlington, Ontario, Canada; 3 University College London, London, UK; 4 Sociedade Brasileira de Nefrologia, São Paulo, Brazil; 5 Escola Bahiana de Medicina e Saúde Pública, Salvador, Brazil; 6 London School of Economics, London, UK; 7 Global Market Access & Pricing, AstraZeneca, Gaithersburg, Maryland, USA

The Author Replies:

We appreciate the insightful editorial letter by Hole *et al.*,^1^ regarding future dialysis needs and the importance of conservative care, in response to our original article outlining the methodology and validation of the IMPACT CKD model—an individual microsimulation model designed to project the holistic burden of chronic kidney disease, using the UK population as a case study.^2^

With an aging UK population, chronic kidney disease cases are expected to increase with progression to end-stage kidney disease (ESKD), placing significant strain on kidney transplant and dialysis services.^3^ Although our model projects an increase in ESKD patients in the UK,^2^ we acknowledge that not all patients with ESKD initiate dialysis.^4^^,^^5^ Accordingly, our model incorporates ESKD management options that were validated by clinical experts. These include remaining in stage 5 while awaiting initiation of kidney replacement therapy, starting dialysis, receiving a kidney transplant, or opting for conservative care instead.^2^ As detailed in the Supplementary Material of our manuscript, these proportions are determined by estimated glomerular filtration rate and patient age.^2^ In addition, as described in the Methods section, our model includes a cap on incident dialysis and a 3% annual growth rate on this cap for the UK, reflecting historical trends and considering health care system constraints.^2^ Of note, the 75% projected growth in patients on dialysis from 2022 to 2032 refers to prevalent patients, not incident cases, with validation outlined in our manuscript.^2^

We share Hole *et al.*’s perspective that conservative care should be used where appropriate either because of limited kidney replacement therapy supply or patient choice, given that it could play a critical role in patient comfort and well-being. Supportive care may be a preferable alternative to dialysis for frail, multimorbid patients because it can alleviate the risks associated with kidney replacement therapy in elderly individuals.^4^^,^^5^ Although supportive care may improve quality of life, it does not extend life expectancy.^4^^,^^5^

We advocate for health policies that prioritize early chronic kidney disease detection and treatment, aiming to reduce the expected increase in progression to ESKD, improve patient survival and quality of life, decrease productivity losses, and minimize the burden on health care systems by mitigating demand for conservative care, transplant, and dialysis.^4^^,^^5^
